# The challenges of classifying big genera such as *Ipomoea*

**DOI:** 10.1002/tax.12887

**Published:** 2023-03-06

**Authors:** Pablo Muñoz-Rodríguez, John R.I. Wood, Tom Wells, Tom Carruthers, Alex Sumadijaya, Robert W. Scotland

**Affiliations:** 1 Department of Biology, University of Oxford, South Parks Road, Oxford, OX1 3RB, United Kingdom; 2 Department of Biodiversity, Ecology and Evolution, Universidad Complutense de Madrid, C/ José Antonio Novais, 12, 28040 Madrid, Spain; 3 Royal Botanic Gardens Kew, Richmond, London, TW9 3AE, United Kingdom; 4 Department of Ecology and Evolutionary Biology, University of Michigan, 1105 North University Ave, Biological Sciences Building, Ann Arbor, Michigan 48109-1085, U.S.A.; 5 Herbarium Bogoriense, Research Center for Biology, National Research and Innovation Agency (BRIN), 23 Cibinong 16911, West Java, Indonesia

**Keywords:** classification, Convolvulaceae, monophyly, systematics, taxonomy

## Abstract

Big genera represent a significant proportion of the world’s plants. However, comprehensive taxonomic and evolutionary studies of these genera are often complicated by their size and geographic spread. This paper explores the challenges faced in classifying these megadiverse plant groups consequent to the existing tension between diagnosability and increasing levels of resolution from molecular sequence data. We use recent examples from across angiosperms to illustrate how monophyly, diagnosability and completeness interplay with each other in attempts to classify several big genera and, specifically, the genus *Ipomoea* (Convolvulaceae). *Ipomoea* and the tribe Ipomoeeae have been the object of recent taxonomic and phylogenetic studies that highlight the limitations of previous attempts to classify the group, and show that the smaller segregate genera traditionally recognised in Ipomoeeae are nested within *Ipomoea* and are neither monophyletic nor diagnosable. We argue that existing classifications must be abandoned, and that recognising an expanded *Ipomoea* that incorporates all segregate genera of the Ipomoeeae is the most appropriate solution as it reconciles the properties of monophyly, diagnosability and completeness, and favours nomenclatural stability.

## Introduction

Approximately 370,000 species of flowering plants have been described to date (RBG Kew, 2016), with 13.5% of these species (c. 50,000 spp.) belonging to the so-called *big genera*. That is, genera that include five hundred or more species, such as *Acacia* Mill., *Allium* L., *Begonia* L., *Carex* L., *Erica* Tourn. ex L., *Eucalyptus* L’Hér., *Euphorbia* L., *Ipomoea* L., *Salvia* L., and *Solanum* L. (Frodin, 2004). Of the c. 13,200 angiosperm genera only around 57 can be considered big genera (cf. Frodin, 2004), yet they constitute an important part of plant diversity and contain plants of economic, horticultural, ecological and cultural significance. Big plant genera are a natural phenomenon akin to big families such as Asteraceae Bercht. & J.Presl, Orchidaceae Juss. or Poaceae Barnhart (Brummitt, 1992; Domínguez Lozano & Schwartz, 2005). Although explanations for the occurrence of species-rich taxa are varied, there is little doubt that they are a feature of all Linnaean classifications, which always include a large number of species-poor taxa together with a small number of species-rich taxa (Willis, 1922; Willis & Yule, 1922; Sanderson & Wojciechowski, 1996; Scotland & Sanderson, 2004; Domínguez Lozano & Schwartz, 2005).

Big genera represent a challenge for taxonomic and evolutionary studies due to their size and geographical spread. Some authors (see historical account in Frodin, 2004: 754–757) refer to these genera as “too big”, a criticism that can only be valid for practical, rather than ontological reasons. The sheer number of specimens in natural history collections from all over the world and the logistics of bringing them together is daunting. Furthermore, the amount of information accumulated over time, the burden of an extensive and widely dispersed literature, and changes in the publication system in recent decades (i.e., impact factor and institutional pressure to publish regularly) have often discouraged researchers from monographing big genera at a global scale. In consequence, there have been very few comprehensive monographs of these genera since the nine-teenth century, when the number of known species in each genus was far fewer. In addition, most big genera are widely distributed (Frodin, 2004; POWO, 2020) and therefore no study based on one country or even one region is likely to account for all the diversity that exists (see as an example *Ipomoea* in Bolivia in fig. 3 in Muñoz-Rodríguez & al., 2019). Furthermore, as we describe in this paper, judgements based on limited material or material from a geographically restricted area inevitably provide an incomplete explanation of the total variation in the genus and often lead to misleading conclusions. As the number of recognised species keeps growing, the study of these big groups becomes even more challenging – although the integration of modern research tools can accelerate the speed at which these studies are conducted (Scotland & Wood, 2012; Muñoz-Rodríguez & al., 2019).

Due to these challenges, taxonomists have frequently tried to break big genera up into more manageable units, either by splitting them into separate genera or by recognising infrageneric taxa, such as subgenera, sections, or series. Until about 25 years ago, these decisions were based on observed morphology, but in recent years molecular systematics has revolutionised the study of evolutionary relationships with implications for generic limits and infrageneric classification, providing more objective grounds for the recognition of genera and infrageneric categories. However, in solving some problems, molecular phylogenetics has created other difficulties, notably through increased phylogenetic resolution that uncovers cryptic nodes, which cannot be correlated with observable morphological synapomorphies (Muñoz-Rodríguez & al., 2022b). In addition, molecular data does not resolve issues of rank, that is, whether a particular clade should be recognised at generic or infrageneric level (Backlund & Bremer, 1998).

### A monographic study of the big genus *Ipomoea*

*Ipomoea* is the largest genus in the family Convolvulaceae Juss. and, with c. 800 species, one of the largest plant genera worldwide (Wood & al., 2020). It has a pantropical distribution and includes herbs, shrubs, vines, lianas and trees. The genus is also present in some more temperate regions as far north as Canada and several widespread species have a world-wide distribution (Wood & al., 2020). Two species have global importance as crops: the sweet potato, *Ipomoea batatas* (L.) Lam., and the kangkong or water spinach, *I. aquatica* Forssk. Similarly, cultivated ornamental species, such as *I. tricolor* Cav. and *I. purpurea* (L.) Roth – the group commonly known as morning glories – and species considered invasive, such as *I. cairica* (L.) Sweet and *I. indica* (Burm.) Merr., are also more or less well known.

The authors of this paper have been working on the systematics of *Ipomoea* since 2012, and in 2014 we embarked on the study of the genus at a global scale. Although hundreds of studies on *Ipomoea* have been published since the genus was validly published (Linnaeus, 1753: 159), it had never been monographed. The only previous “global” taxonomic review, published by Choisy more than 170 years ago, included just 282 species (Choisy, 1834, 1838, 1845), and more recent studies focused on small parts of the genus (e.g., Marderosian, 1965; Austin, 1991; Staples & al., 2005; Ogunwenmo, 2008; Austin, 2013; Austin & McDonald, 2014; Swamy & Ramana, 2018; Rattanakrajang & al., 2022) or on small groups of species, frequently those related to sweet potato or other widespread species (McDonald & Austin, 1990; Austin & Staples, 1991; Austin, 1997; Das, 2011; Abdel Khalik & al., 2012; Abdel Khalik, 2013; Folorunso, 2013). A few authors also addressed the genus at a national or regional level, but several of these more extensive treatments date back to the 19th century or the first half of the 20th century (Grisebach, 1864; Gray, 1878; Hallier, 1894; House, 1908) and most of the more recent, with a few notable exceptions (e.g., Van Ooststroom, 1953; O’Donell, 1960; Verdcourt, 1963; Austin, 1975, 1982; Meeuse & Welman, 2000; Wood & al., 2015), are basic checklists of species or provide only limited information about the taxa included. *Ipomoea* species new to science are still described regularly, especially from Brazil, Mexico, India and Southeast Asia (e.g., Traiperm & al., 2019; Santos & al., 2020, 2021; Lawand & Shimpale, 2021), although authors often do not discuss their discoveries in a broader taxonomic context.

A milestone of our work was the publication of a taxonomic monograph of all 425 *Ipomoea* species in the Americas (Wood & al., 2020) alongside comprehensive molecular phylogenies of the tribe Ipomoeeae Hallier f. (Muñoz-Rodríguez & al., 2019). The results of our monographic work also include the description of over 70 American species new to science – almost 9% of all species known worldwide –, an extensive nomenclatural review, and several other taxonomic publications (Wood & al., 2015, 2016a,b, 2017a,b,c, 2018; Wood & Scotland, 2017a,b,c; Jara & al., 2020; Muñoz-Rodríguez & al., 2019, 2022a). The molecular phylogenies also allowed us to address the evolution of the genus at a global scale (Muñoz-Rodríguez & al., 2018, 2019, 2022a,b; Carruthers & al., 2020a). Our results are supported by the study of over 25,000 physical herbarium specimens, with visits to herbaria in the U.K. and other European countries, Argentina, Bolivia, Brazil, Colombia, Cuba, Ecuador, Paraguay, Peru, United States and specimens in virtual herbaria, as well as fieldwork in Argentina, Bolivia, Brazil, Ecuador, and Paraguay. Our molecular phylogenies currently include c. 60% of all species in tribe Ipomoeeae, with 2000 specimens sequenced for DNA barcodes corroborated for accuracy against whole chloroplast genomes and 384 putative single-copy nuclear regions from c. 400 samples representing 215 species.

### Taxonomic implications for *Ipomoea* and Ipomoeeae

**Fig. 1:**
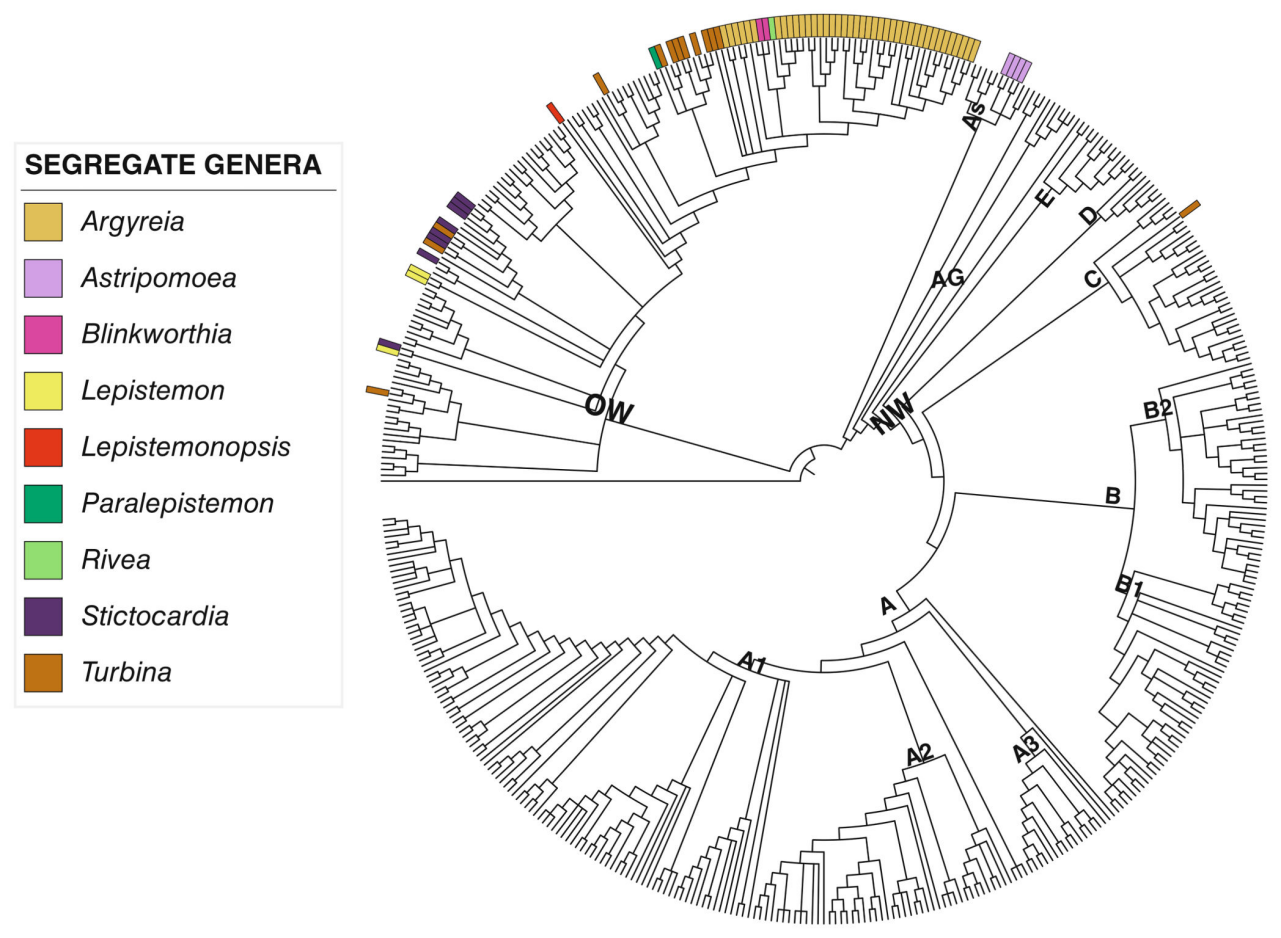
The taxonomy of Ipomoeeae has been constantly revised since the 18th century, and many species have been classified in different genera by different authors. This nrITS phylogeny of Ipomoeeae, modified from Muñoz-Rodríguez & al., 2019, includes c. 60% of all species in the tribe, with one specimen per species; it summarises the current taxonomic situation, with all recognised segregate genera nested within *Ipomoea*. Colour bars indicate species currently classified in a segregate genus. Only the segregate genus *Astripomoea* (purple) forms a small, monophyletic group within the Astripomoea clade (As), with all other segregate genera polyphyletic. An important point about the Old World clade is that it contains more species of *Ipomoea* (c. 250) than all segregate genera combined (c. 170). NW = New World clade; OW = Old World clade; AG = African grade; A = clade A; A1 = Carnea clade; A2 = Digitata clade; A3 = Batatas clade; B = clade B; B1 = Purpurea clade; B2 = Quamoclit clade; C = Pes-caprae clade; D = Squamosa clade; E = Cairica clade; As = Astripomoea clade. An expanded phylogeny with all labels is provided in suppl. Fig. S1. This nrITS phylogeny is for illustrative purposes only as its usefulness in taxonomic studies is always in tandem with morphological species hypotheses and high-throughput nuclear and chloroplast phylogenies. See Muñoz-Rodríguez & al. (2019) for a full explanation and an expanded, multi-specimen phylogeny.

*Ipomoea* has been traditionally classified in the tribe Ipomoeeae alongside several smaller segregate genera such as *Argyreia Lour., *Astripomoea A.Meeuse, *Batatas* Choisy, *Blinkworthia Choisy, *Calonyction* Choisy, *Calycanthemum* Klotzsch, *Exogonium* Choisy, *Lepistemon Blume, *Lepiste-monopsis Dammer, *Mina* Cerv., *Paralepistemon Lejoly & Lisowski, *Quamoclit* Mill., *Rivea Choisy, *Stictocardia Hallier f., or *Turbina Raf. These smaller genera have been recognised by different authors throughout history, with most of them later sunk into *Ipomoea* and only a handful still recognised at present (indicated with an asterisk above). In 2019, our molecular phylogenies (Muñoz-Rodríguez & al., 2019) confirmed that all the smaller genera in Ipomoeeae are nested within *Ipomoea* and all but one of them (*Astripomoea*) are non-monophyletic (Fig. 1, suppl. Fig. S1). This had been shown several times before, albeit with a less comprehensive taxon and data sampling (see for example Miller & al., 1999; Wilkin, 1999; Stefanović & al., 2003; Eserman & al., 2014). In addition, we also demonstrated that the three subgenera traditionally recognised within *Ipomoea*, and almost all sections and series, were not monophyletic (suppl. Fig. S2) (Muñoz-Rodríguez, 2019; Muñoz-Rodríguez & al., 2019, 2022b). Thus in 2019, considering all evidence available, we embraced the idea, first suggested by Paul Wilkin (1999) and reiterated by others (e.g., Stefanović & al., 2003), of recognising an expanded *Ipomoea* that includes all other genera in Ipomoeeae. Members of this expanded, monophyletic genus *Ipomoea* can be recognised by their spiny pollen, a morphological synapomorphy within Convolvulaceae (all other members of the family have smooth pollen exine). We also undertook the necessary nomenclatural changes to recognise an expanded *Ipomoea* that contained all species previously included in other genera (supplementary information in Muñoz-Rodríguez & al., 2019). This decision, however, has been questioned by colleagues who prefer to continue using the smaller non-monophyletic genera aforementioned and a non-monophyletic *Ipomoea* (Traiperm & Suddee, 2020; Lawand & Shimpale, 2021; Staples & al., 2021; Rattanakrajang, & al. 2022).

In this paper, we discuss how we reached this and other decisions in our work on *Ipomoea*. We structure the paper in three main sections. First, we present the theoretical framework that we think is relevant to a discussion on biological classifications based on four criteria: monophyly, resolution, diagnosability, and completeness. Second, we use recent examples from across angiosperms to illustrate how these different criteria interplay with each other, with special attention to other big genera, and explore the approaches taken by researchers in these groups. Finally, we discuss the case of *Ipomoea* and several alternatives for its classification.

## Theoretical Framework

**Fig. 2:**
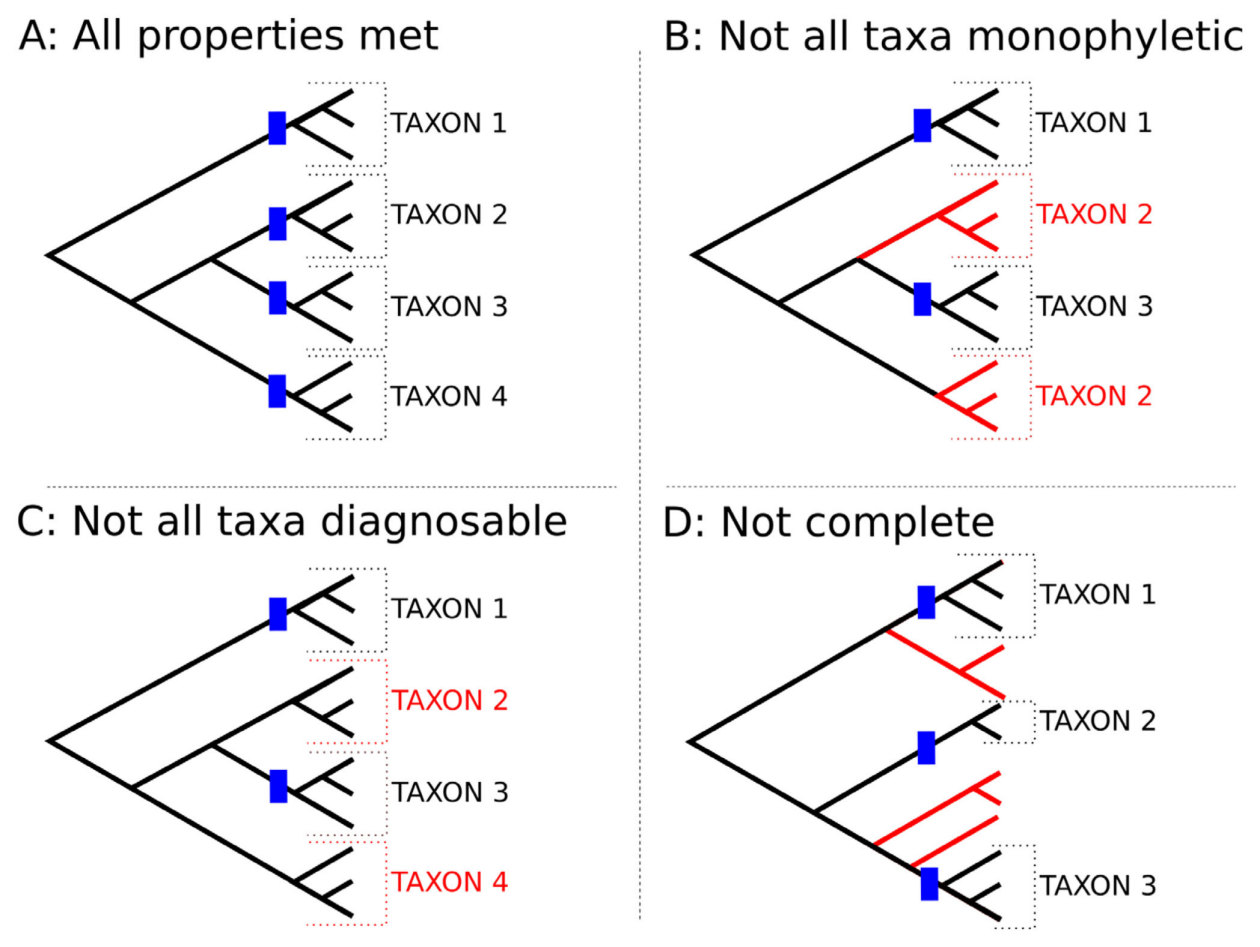
**A**, In an ideal classification of any group of organisms, all taxa should be monophyletic (dashed lines) and diagnosable (blue boxes), and no taxon should be left unplaced. **B**, Often, however, one or more taxa are not monophyletic but split into two or more distinct lineages more closely related to other taxa (e.g., taxon 2). **C**, Many classifications include formally recognised groups that are monophyletic but do not present diagnosable morphological characters, and thus can only be identified using molecular data. This is unsatisfactory as at present it is not possible to sequence every specimen studied. **D**, It is common that classifications fail to account for all taxa (e.g., species) in the group, leaving some of them unplaced. The lack of a taxonomic context hinders the study of these species in the right framework.

A review of recent papers on the classification of big genera suggests four criteria are considered as important by most authors: *monophyly, resolution, diagnosability*, and *completeness*. These four criteria are an implicit feature of all classifications (Fig. 2A).

First, it is now widely accepted that classifications should reflect evolutionary history and a taxon should include all descendants of a common ancestor (Hennig, 1966). Thus, all taxa should be *monophyletic*. Monophyly was accepted by many, more than 20 years ago, as the overarching principle for classification, with other properties subordinated to it (Bryant, 1994; Backlund & Bremer, 1998) – although there does seem to have been an ongoing, stubborn resistance to this idea in the taxonomic literature (Brummitt, 1996; Hörandl & Stuessy, 2010). The recognition of non-monophyletic taxa, e.g., polyphyletic sections or series (Fig. 2B), hinders the interpretation of biological observations in an evolutionary context.

The second property, *resolution*, is tightly linked with monophyly. Lack of resolution may hinder recognition of the existing breadth of phylogenetic diversity (Swenson, 2009). Although poorly resolved phylogenies exist, the exponential increase in genomic data generation has made high levels of phylogenetic resolution a realistic goal (Muñoz-Rodríguez & al., 2022b).

Third, to be most useful, all taxa in a classification should be *diagnosable* by a morphological character or a combination of characters. In the best-case scenario, morphological diagnosability is also supported by geographical distribution and behaviour/ecology. Even when diagnosability is considered secondary to monophyly, a classification that recognises, for example, subgenera that are monophyletic but lack diagnostic characters (Fig. 2C) would in practice be of limited use, as it would not be possible to place specimens in their evolutionary context unless using molecular data. Given that most plant species have never been sequenced (Sayers & al., 2021) it would result in many unplaced specimens and species, also affecting completeness.

Fourth, a Linnaean classification should be *complete*, that is, it should account for all species in a group and not leave unplaced species. If a genus is divided, for example, into three subgenera, every species in the genus is ideally assigned to one of them. A classification that leaves a rump of unassigned species (Fig. 2D) is inherently incomplete, unsatisfactory and intrinsically problematic for other users.

An additional criterion, not theoretical but pragmatic and desirable is *nomenclatural stability* (Carruthers & Robin, 2010; Wright, 2015: 125; Drew & al., 2017). Many large genera are of economic or horticultural importance and names are used in commercial and cultural settings as well as by ecologists, conservationists, horticulturalists, etc. Users welcome nomenclatural stability and this is something that should be aimed for where possible. Many of these users are resistant to change as evidenced by the persistence of long abandoned names such as “azalea” (syn. *Rhododendron* L.) in horticultural settings. However, we think nomenclatural stability should in no case overrule monophyly. In fact, the issue of stability can be overstated as for example the sinking and nomenclatural changes of *Lycopersicon* Mill. into *Solanum* and *Rosmarinus* L. into *Salvia* seem to have been readily accepted (Drew & al., 2017; Gagnon & al., 2022).

## Reconciling Theory and Practice In Big Plant Genera

The criteria for a classification outlined above are relatively uncontroversial. However, taxonomists often find difficulties in reconciling them, and in practice a constant tension exists between the application of all four properties. This tension can lead to conflict between, for example, monophyly and diagnosability. In such cases, taxonomists often prioritise one at the expense of the other with the consequent recognition of either non-monophyletic or non-diagnosable taxa.

In this section we review how recent decisions have been made in other genera to put our proposal for *Ipomoea* in a broader context. We specifically focus on five other megadiverse genera (*Carex, Solanum, Salvia, Euphorbia, Begonia*) and discuss these taxonomic decisions relative to the general properties of classification discussed above.

### Case study 1. *Carex*

*Carex* is the largest genus in the family Cyperaceae Juss. It is classified in tribe Cariceae Dumort., which includes c. 2000 species traditionally divided into five genera: *Carex* (>1830 spp.), *Uncinia* Pers. (c. 70 spp.), *Kobresia* Willd. (c. 60 spp.), *Schoenoxiphium* Nees (c. 15 spp.), and *Cymophyllus* Mack. ex Britton & A.Br. (monotypic). Molecular phylogenetics have confirmed that the tribe Cariceae is a monophyletic group but *Carex* and *Kobresia* as traditionally recognised are polyphyletic, and *Schoenoxiphium* and *Uncinia*, although monophyletic, are nested within *Carex* (Global Carex Group, 2015 and references therein).

As explained by the Global Carex Group (2015), a new classification that better reflected evolutionary relationships was needed. *Carex* specialists thus considered three possibilities:

(1) To treat the four major clades as four distinct genera.
(2) To recognise each strongly supported clade within Cariceae as a distinct genus.
(3) To recognise the entire tribe Cariceae as a single genus, *Carex*.

Option 1 was ruled out due to lack of diagnosability. Although three of the four major clades have strong molecular support, the morphological variation makes it difficult to define synapomorphies. In turn, option 2 was ruled out because it would imply extensive nomenclatural rearrangements, and because some of the putative genera would be morphologically very similar and this would cause considerable confusion. The Global Carex Group therefore opted for option 3, to recognise an expanded, monophyletic *Carex* as the only genus in tribe Cariceae. An expanded *Carex* is *monophyletic, diagnosable* (its unisexual flowers and the perigynia surrounding the female flowers are synapomorphies within the family), and *complete* (accounts for all species), and involves fewer name changes than the other options, causing less potential disturbance to other users. In addition, it is important to note that authors do not seem to be concerned with the size of the genus – more than twice the size of an expanded *Ipomoea*.

### Case study 2. *Solanum*

*Solanum* is the largest genus in the family Solanaceae Juss. Prior to molecular studies, the traditional definition of *Solanum* excluded taxa with stamen dimorphism and/or anther modifications and treated them as different genera, e.g., *Cyphomandra* Mart. ex Sendtn., *Lyco-persicon* Mill., and *Normania* Lowe. Over the past decades, the use of DNA sequence data showed that these genera were nested within *Solanum* (Spooner & al., 1993; Bohs & Olmstead, 1997; Olmstead & Palmer, 1997) and were subsequently sunk into it. The changes in the generic circumscription of *Solanum* have had a minimal effect on its size but have expanded its morphological diversity, especially in relation to anther characteristics.

*Solanum* presents a relatively uniform floral morphology. In terms of an infrageneric classification, and prior to molecular studies, sections were defined based on morphological characters (Hunziker, 2001), but most of these have later been shown to be non-monophyletic (Olmstead & Palmer, 1997; Weese & Bohs, 2007). Following a proposal by Bohs (2005), *Solanum* researchers have used informal names to refer to clades within *Solanum* (see fig. 1 in Särkinen & al., 2013) and no formal infrageneric classification has been attempted. Some of the clades have diagnostic morphological characters, whereas other clades do not. Furthermore, the complex nomenclature and concepts used across *Solanum*, as well as the possible existence of hard polytomies along the backbone of the genus (Gagnon & al., 2022) suggest that any attempt to classify *Solanum* into formal sections would add to the confusion more than facilitate understanding (Tiina Särkinen, pers. comm.).

### Case study 3. *Salvia*

*Salvia*, as traditionally recognised (Drew & al., 2017; Mabberley, 2017) is an almost-cosmopolitan genus only absent from Australia and New Zealand. It forms a morphologically homogeneous group readily identifiable by the staminal lever mechanism (Sprengel, 1793; Claßen-Bockhoff & al., 2004). However, it has been shown that *Salvia* in its current delimitation is not monophyletic (Drew & al., 2017 and references therein), and that the lever mechanism appeared at least three times independently – or, alternatively, the lever mechanism was the ancestral character state and was subsequently lost multiple times in different lineages (Walker & Sytsma, 2007; fig. 2 in Drew & al., 2017). Interestingly, the five lineages that do not present the staminal lever mechanism correspond to five segregate genera traditionally recognised in Lamiaceae: *Dorystaechas* Boiss. & Heldr. ex Benth., *Meriandra* Benth., *Perovskia* Kar., *Rosmarinus* L., and *Zhumeria* Rech.f. & Wendelbo. These five genera together amount to 15 species, a small number in comparison with *Salvia*.

Similar to the case in *Carex*, three possibilities were considered to deal with the non-monophyly of *Salvia* (Walker & al., 2004):

(1) To treat all different lineages within *Salvia* sensu lato as different genera. This option was supported, for example, by Will & al. (2015), who argued in favour of recognising the segregate genera and splitting *Salvia* into smaller groups. This approach would imply extensive nomenclatural rearrangements as the type of *Salvia, S. officinalis* L., belongs to a clade of c. 250 species that would retain the name *Salvia*, whereas over 500 other species would be transferred to other genera – including all but eight species in the Americas.
(2) To employ phylogenetic nomenclature (the Phylo-Code), a rank-free system of biological nomenclature (De Queiroz & Gauthier, 1992; Cantino & al., 2020), and retain current species binomials while naming the different clades. This was proposed in the late 20th century for mints and their relatives (Cantino & al., 1997) but has not been followed in recent publications.
(3) To keep the genus *Salvia* as traditionally circumscribed while treating the five segregate genera as part of it (Drew & al., 2017). This approach would achieve monophyly while only requiring minor nomenclatural rearrangements to accommodate the c. 15 species transferred to *Salvia* and seems to be gaining acceptance (e.g., González-Gallegos & al., 2020). On the other hand, sinking the five segregate genera within *Salvia* comes at the cost of rendering the lever mechanism found in *Salvia* s.str. no longer diagnostic for *Salvia* s.l. At the moment, no formal or comprehensive infrageneric classification of *Salvia* has been proposed, and researchers use informal names to refer to clades within an expanded *Salvia* that are distinguished geographically and/or morphologically (Jenks & al., 2013; Fragoso-Martínez & al., 2018), akin to the approach used in *Solanum*.

### Case study 4. *Euphorbia*

With over 2000 accepted species, *Euphorbia* is the second-largest genus of flowering plants after *Astragalus* L. *Euphorbia* species are characterised by the cyathium, a morphological synapomorphy. In 2002, phylogenies using nuclear and chloroplast DNA sequence data and around 220 species showed that former segregate genera such as *Chamaesyce* Gray, *Monadenium* Pax, *Pedilanthus* Neck. ex Poit., and *Poinsettia* Graham were nested within *Euphorbia*, and were thus synonymised into it with little conflict (Steinmann & Porter, 2002). The data also showed a marked incongruence with most of the traditionally recognised subgeneric groups within *Euphorbia*, clearly indicating that previous subgenera and sections delimited based on morphology are not monophyletic. Subsequently, broadly sampled studies have clarified the relationships between *Euphorbia* s.l. (i.e., including all formerly segregate genera) and other groups within Euphorbiaceae Juss., clearly showing that all segregate genera with a cyathium are embedded within *Euphorbia* (Steinmann & Porter, 2002; Wurdack & al., 2005; Bruyns & al., 2006; Horn& al., 2012). These studies have also identified four main clades within *Euphorbia* with no apparent morphological synapomorphies. These clades have been recognised as four distinct subgenera: *E*. subg. *Euphorbia* (c. 660 spp., Dorsey & al., 2013), subg. *Chamaesyce* Raf. (c. 600 spp., Yang & al., 2012), subg. *Esula* Pers. (c. 480 spp., Riina & al., 2013), and subg. *Athymalus* Neck. ex Rchb. (c. 150 spp., Peirson & al., 2013).

### Case study 5. *Begonia*

*Begonia* L. is one of the fastest-growing genera of angiosperms in terms of number of species described, with over 700 new species names published in the last two decades (IPNI, 2021). *Begonia* as currently delimited has a near-pantropical distribution but is absent from Australia, and species often have restricted distributions (c.f. Doorenbos & al., 1998; Forrest & Hollingsworth, 2003). It is one of only two recognised genera in the family Begonia-ceae C.Agardh, the other one being monotypic, *Hillebrandia* Oliv. (POWO, 2020). An alternative approach would be to split *Begonia* into multiple genera with the recognition of monophyletic groups at generic level. This approach, however, would be extremely disruptive to the nomenclature of this otherwise easy-to-recognise, horticulturally important group, as only the American species closely related to the type species (*B. obliqua* L.) would retain the name *Begonia*. Researchers working on *Begonia* have thus opted for an expanded, monophyletic *Begonia* with a sectional classification of the genus (Doorenbos & al., 1998; Moonlight & al., 2018). A sectional classification has the advantage over a generic classification that taxonomic instability does not produce nomenclatural instability, as species can be moved between sections with no impact on their generic name or authority string (Turland & al., 2018).

A complication of this sectional classification for *Begonia* studies, however, is that many of the sections traditionally recognised based on morphology (Doorenbos & al., 1998) are not monophyletic (see fig. 2 in Moonlight & al., 2018). In addition, this classification was incomplete as 80 species were only tentatively assigned to section and some 50 species were not assigned at all. The current sectional classification of *Begonia* thus needs updating to achieve a natural (monophyletic) sectional classification informed by morphology (Peter Moonlight, pers. comm.).

### Conflict between monophyly and diagnosability is recurrent

Conflict between monophyly and diagnosability is recurrent in angiosperm genera. It is sometimes not easily settled as in the case of *Phyllanthus* L. (Phyllanthaceae Martinov), a widespread genus with c. 875 species and a complex taxonomic history. As traditionally circumscribed, *Phyllanthus* is paraphyletic, with three smaller genera nested within: *Breynia* J.R.Forst. & G.Forst. (c. 90 spp.), *Glochidion* J.R.Forst & G.Forst. (c. 300 spp.), and *Synostemon* F.Muell. (c. 30 spp.) (Bouman & al., 2021). However, while it is clear that *Phyllanthus* as traditionally circumscribed is not monophyletic, a general agreement on how to delimit it has not been reached. Two main solutions have been proposed, either to recognise an expanded, monophyletic albeit morphologically heterogeneous *Phyllanthus* (Kathriarachchi & al., 2005; Hoffmann & al., 2006) or to divide the tribe Phyllantheae into multiple monophyletic genera (Pruesapan & al., 2008; Van Welzen & al., 2014; Bouman & al., 2021). The most recent publication (Bouman & al., 2021) argued that an expanded *Phyllanthus* would be difficult to define, and thus the authors recommended splitting *Phyllanthus* into nine distinct mono-phyletic genera. However, diagnosability issues persist, as the authors do not identify morphological characters to diagnose these genera.

Uncertainty is not limited to big genera. Bruyns & al. (2017), for example, proposed the recognition of an expanded, monophyletic genus *Ceropegia* L. to include *Brachystelma* R.Br. and the approximately 30 other genera of Apocynaceae Juss. known as the stapeliads that are nested within *Ceropegia*. However, some authors still prefer to differentiate *Ceropegia* and *Brachystelma* based on their distinct floral morphologies, prioritising diagnosability over monophyly (Rasingam & Swamy, 2020): several new species of *Brachystelma* have been described in recent years, and the name *Brachystelma* is still used in horticultural settings. Finally, other authors argue the stapeliads, although nested within *Ceropegia*, form a morphologically well-defined group that “makes a strong case for the acceptance of paraphyletic groupings” (Gilbert, 2020). Gilbert (2020) also argues that splitting *Ceropegia* into multiple new genera would cause a major disruption in data connectivity and would serve little useful purpose.

A final example is the relationship between *Euploca* Nutt., *Heliotropium* Tourn. ex L., and *Myriopus* Small, three genera in the family Heliotropiaceae sensu APG IV (Stevens, 2001–). In this case, monophyly is deemed more important than diagnosability: the three genera have been recently delimited by Frohlich & al. (2022) as reciprocally monophyletic even though there are no clear diagnostic characters to differentiate them.

In summary, conflict between monophyly and diagnosability is widespread in many angiosperm genera, regardless of their size, and the incorporation of molecular analysis to not-yet-studied groups is likely to exacerbate the conflict. Researchers working on big genera (>800 spp.) have faced similar challenges as those faced now by *Ipomoea* researchers. They have attempted to reconcile the theoretical criteria of an ideal biological classification (monophyly, diagnosability, completeness) while aiming at nomenclatural stability. It must be clear, nevertheless, that all decisions imply nomenclatural rearrangements, from making just 15 new combinations in the case of *Salvia* to changing hundreds of names in *Phyllanthus* and over 150 names in *Carex*. In most cases, the incorporation of molecular data has revealed that small, segregate genera are nested within larger ones, and that many infrageneric ranks are paraphyletic or polyphyletic, subsequently leading to alternative proposals to redefine generic and infrageneric boundaries. In all big genera discussed above authors favoured monophyly, either by splitting a paraphyletic genus into smaller, monophyletic genera (e.g., *Phyllanthus*) or by expanding the larger genus to incorporate all formerly recognised smaller segregates (e.g., *Begonia, Carex, Euphorbia, Salvia, Solanum*).

Diagnosability has long been an implicit requirement in taxonomic studies as exemplified in the cases above and in the use of morphological diagnoses and identification keys. However, the recognition of cryptic nodes is becoming more common as a consequence of increasing levels of phylogenetic resolution (Fazekas & al., 2009; Muñoz-Rodríguez & al., 2022b). Nevertheless, even when cryptic nodes can be identified through molecular methods, a classification that recognises them may be of little practical value as such nodes cannot be identified except as a result of sequencing in a laboratory, while most plants have never been sequenced, even for a single DNA barcode region (Sayers & al., 2021). In conclusion, we consider a classification of flowering plants that does not attempt a degree of morphological diagnosability is neither ideal nor functional and so is of limited value.

### Completeness

Historically most taxonomists have followed the principle of completeness, leaving only a short appendix in taxonomic treatments with a few unplaced species. There have been exceptions; Bremekamp’s monograph of the Acanthaceae subtribe Strobilanthinae (Bremekamp, 1944), for example, recognised 52 genera – mostly splits from *Strobilanthes* Blume – but failed to account for around 100 species previously placed in *Strobilanthes* (c. 30% of the total number of species in the genus). Consequently, herbaria, floras and checklists had to account for two kinds of *Strobilanthes*: a small, supposedly monophyletic group of species separated off by Bremekamp, and a large polyphyletic group of miscellaneous species with no close connection to *Strobilanthes* sensu stricto (Carine & Scotland, 2002).

Completeness problems were a feature of the traditional circumscription of *Ipomoea*. Authors recognised three subgenera – *I*. subg. *Ipomoea*, subg. *Eriospermum* Verdc., and subg. *Quamoclit* (Moench) C.B.Clarke – but these only included a fraction of all species known in the genus, leaving many species unclassified (suppl. Fig. S2) (Muñoz-Rodríguez, 2019, 2022b). This is not just a historical issue but contemporary as in the case of *Begonia* discussed above. Biological classifications should aim to include all species in the group with no significant residue of unassigned species.

## Moving Forward: Alternatives For A Classification Of Ipomoeeae

There are three options for a classification of Ipomoeeae: (1) to continue using the current system, (2) to attempt a new classification with multiple genera newly defined, or (3) to recognise an expanded genus *Ipomoea*. In the rest of this paper, we discuss these different possibilities in our current state of knowledge and in the context of the theoretical framework outlined above.

### Option 1. Business-as-usual

The first option is to continue using the current system; that is, to recognise segregate genera, such as *Argyreia, Lepistemon* and *Rivea*, as in recent publications (Traiperm & Suddee, 2020; Lawand & Shimpale, 2021; Staples & al., 2021; Rattanakrajang & al., 2022). Since all smaller genera currently recognised except *Turbina* are in what Muñoz-Rodríguez & al. (2019) termed the Old World Clade (Fig. 1), it may seem that they are dominant in that clade and thus this approach would be straightforward. However, that is not the case as the Old World clade contains more species of *Ipomoea* in its traditional delimitation (c. 250) than the number of species of all other genera combined (c. 170).

In addition, it has been repeatedly shown that *Ipomoea* is paraphyletic with all other genera nested inside, and all of these except *Astripomoea* in turn are polyphyletic and intermingle with *Ipomoea* species (Muñoz-Rodríguez & al., 2019) (Fig. 1, suppl. Fig. S1). The significance of many species of *Ipomoea* intermingled with the non-monophyletic segregate genera is that it classifies potential sister species or very closely related species in different genera, and therefore prevents the interpretation of biological and ecological observations in the correct evolutionary framework.

Some recent authors (e.g., Traiperm & Suddee, 2020; Lawand & Shimpale, 2021; Staples & al., 2021; Rattanakrajang & al., 2022) have ignored the issue of monophyly, arguing that the main reason to continue the recognition of these segregate genera is that they can be morphologically distinguished from *Ipomoea*. To avoid unnecessary repetition, we refer the reader to the introductory pages of our monograph of *Ipomoea* for an extensive discussion of the heterogeneous and homoplastic morphology of the group (Wood & al., 2020: 31–53). Nevertheless, just as an example, Lawand & Shimpale (2021: 18–19) claim that the genus *Argyreia* can be distinguished “by an array of characters such as their habit as mostly semi-woody lianas, corolla with hairy midpetaline bands, flowers subtended with well-developed bracts, and an indehiscent berry”. A broader view of the morphology of Ipomoeeae shows these morphological characters are not restricted to *Argyreia* but appear commonly elsewhere in Ipomoeeae, often in distant parts of the phylogeny. The woody or semi-woody liana habit of *Argyreia* species appears also, for example, in clades A1 (e.g., *Ipomoea bombycina* (Choisy) Benth. & Hook.f. ex Hemsl., *I. populina* House), A2 (e.g., *I. cuprinacoma* E.Carranza & J.A.McDonald, *I. horsfalliae* Hook., *I. schulziana* O’Donell), and Old World (e.g., *I. corymbosa* (L.) Roth ex Roem. & Schult. [= *Turbina corymbosa* (L.) Raf.], *I. fissifolia* (McPherson) Eckenwalder, *I. tiliifolia* (Desr.) Roem. & Schult. [= *Stictocardia tiliifolia* (Desr.) Hallier f.]) (Wood & al., 2020); indehiscent fruits in clade C (e.g., *Ipomoea leptophylla* Torr.) and all species formerly recognised in *Stictocardia* in clade Old World (Miller & al., 1999); corollas with hairy midpetaline bands in clades A (e.g., *I. bracteolata* R.W.Johnson, *I. saintronanensis* R.W.Johnson) and Old World (e.g., *I. cambodiensis* Gagnep. & Courchet, *I. corrugata* Thulin, *I. pes-tigridis* L., *I. plebeia* R.Br.) (Van Ooststroom, 1940; Johnson, 1986; Thulin, 2003; Staples & al., 2014); and flowers subtended with well-developed bracts in clades B1 (e.g., *I. neurocephala* Hallier f.), B2 (e.g., *I. suffulta* (Kunth) G.Don), or Old World (e.g., *I. involucrata* P.Beauv.) (Wood & al., 2020). Furthermore, it is important to note that the multiple origin of some of these morphological characters was reported decades ago. Manos & al. (2001), for example, showed that indehiscent fruits have evolved at least seven times independently in Ipomoeeae, appearing in species of *Argyreia, Stictocardia*, the former *Turbina*, and *Ipomoea* (e.g., *I. aquatica* or *I. leptophylla* Torr.).

It has also been argued that *Argyreia* can be distinguished based on cytology (Lawand & Shimpale, 2021). Cytological studies are few and, in general, use a very limited taxon sampling, which complicates comparisons. Most cytological studies of Ipomoeeae species focused on the same set of species and only a few *Argyreia* species have been analysed, normally 1–3 species per study. In general, studies that focus on chromosome morphology or chromosome length highlight the intra- and interspecific diversity existing in Convolvulaceae and Ipomoeeae, and species of different genera are frequently classified in the same type (e.g., Sampathkumar, 1979). In addition, most species of Ipomoeeae have a basic chromosome number of *n* = 15, while exceptions (e.g., *n* = 14) have only been reported sporadically and in both the segregate genera and in *Ipomoea* (Ting & Kehr, 1953; Ting & al., 1957; Vij & al., 1977). In summary, to the best of our knowledge there is no evidence that the segregate genera in Ipomoeeae have sufficient and consistent cytological differences for the purpose of classification.

Finally, Staples & Traiperm (2017) noted that “In ecological terms, *Argyreia* in Asia seems to have taken over the role filled by *Ipomoea* species elsewhere in the tropics: the two genera appear to be ecological analogues.” This view is misleading as it suggests two separate groups with similar ecological roles exist in different parts of the world, whereas from the phylogeny we know there is only one pantropical group fulfilling this ecological role.

In conclusion, there is no reason to continue using the current classification system with recognition of multiple non-monophyletic genera. Justifications for this “business-asusual” approach are illogical and this system should be abandoned. The claim by some authors (e.g., Traiperm & Suddee, 2020; Lawand & Shimpale, 2021; Staples & al., 2021; Rattanakrajang & al., 2022) that we have not offered enough evidence to abandon this “business-as-usual” approach ignores the fact that Muñoz-Rodríguez & al. (2019, 2022b), Wood & al. (2020) and related publications constitute the most comprehensive study in terms of taxon and character sampling in the history of *Ipomoea* research.

### Option 2. Propose a new classification of Ipomoeeae

**Fig. 3:**
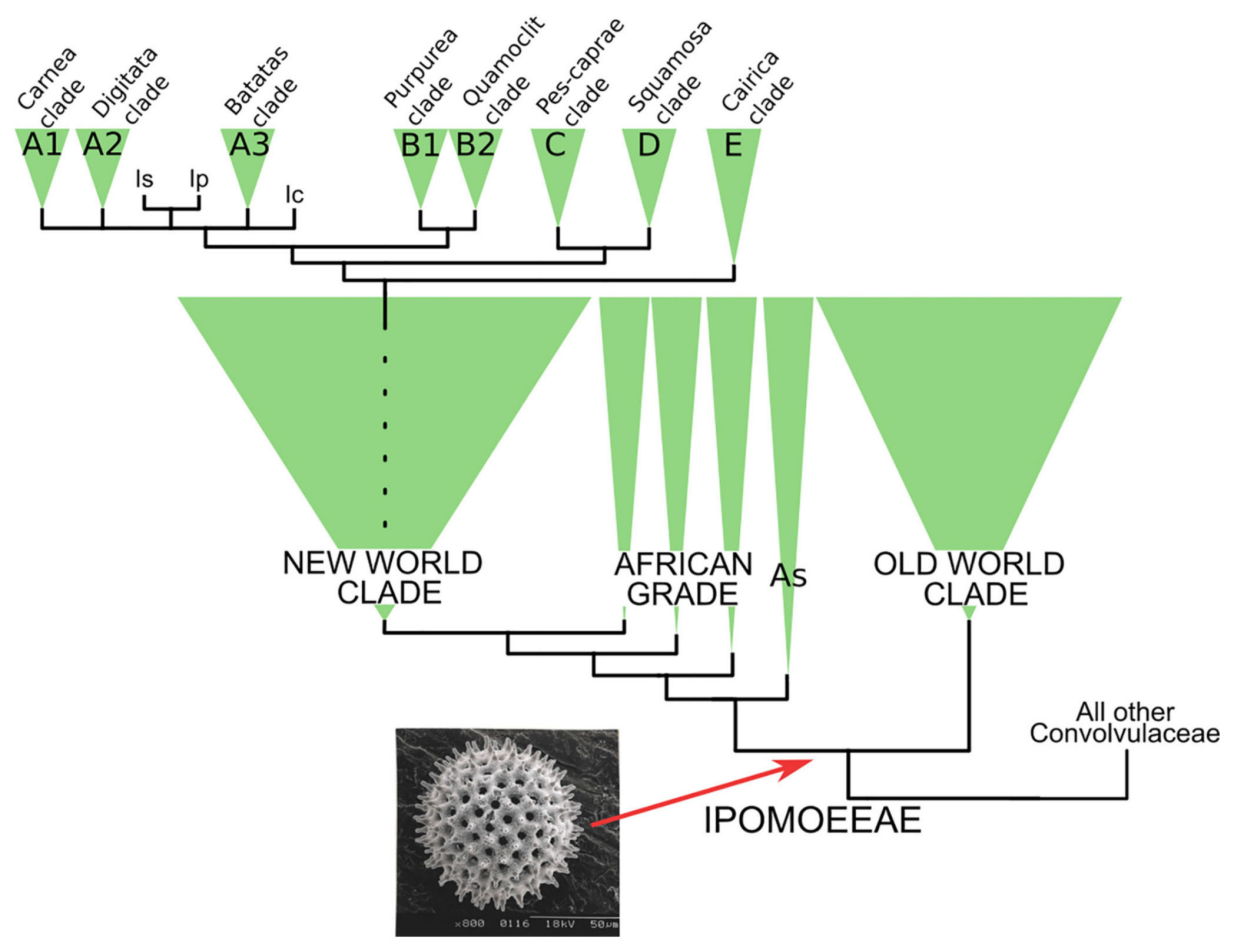
Summary nuclear phylogeny of Convolvulaceae tribe Ipomoeeae. All segregate genera are nested within clades dominated by *Ipomoea* species (in green). All clades shown have 100% support in molecular phylogenies inferred using nuclear and chloroplast data; relationship between A1, A2 and A3, and between B1 and B2 varies for nuclear and chloroplast data (see Muñoz-Rodríguez & al., 2019). New World and Old World clades have roughly the same number of species (c. 400), whereas the African grade includes c. 20 species divided into three clades. The Old World clade includes more *Ipomoea* species than species of all segregate genera combined. The tribe Ipomoeeae is characterised by the echinate pantoporate pollen. A1 = Carnea clade; A2 = Digitata clade; A3 = Batatas clade; B1 = Purpurea clade; B2 = Quamoclit clade; C = Pes-caprae clade; D = Squamosa clade; E = Cairica clade; As = Astripomoea clade; Is = *Ipomoea setosa*; Ip = *I. peruviana*; Ic = *I. cryptica*.

A second possibility is to redefine the groups within Ipomoeeae and delimit new taxa at the same rank, in a generic or infrageneric classification, based on the monophyletic groups Muñoz-Rodríguez & al. (2019) have identified (New World clade, Old World clade, clades A–E; Fig. 3). Whilst this option may be attractive, it would face a problem as significant as the lack of monophyly in the current classification: diagnosable clades in *Ipomoea* are the exception and not the rule, and most of these large clades are not diagnosable (Wood & al., 2020). Clade A (part of the bigger New World clade), for example, includes a quarter of all species of *Ipomoea* world-wide and is further divided into three strongly supported smaller clades: two species-rich clades (clades A1 and A2, with c. 130 and c. 90 species respectively) and clade A3, which includes sweet potato and its 16 close wild relatives. In addition, at least three other species form independent lineages within clade A: *Ipomoea cryptica* J.R.I.Wood & Scotland, *I. peru-viana* O’Donell and *I. setosa* Ker Gawl (suppl. Fig. S1), and chloroplast and nuclear phylogenies resolve different topologies between clades A1, A2, A3 and these three species. Furthermore, this part of the tree (clades A1 and A2) includes two species-rich radiations (Muñoz-Rodríguez & al., 2019; Carruthers & al., 2020a). These radiations are characterised by constant shifts between biomes and growth habits, have no diagnostic characters, and there are no clear-cut boundaries to decide which species are or are not part of the radiation. Monophyletic diagnosable groups in clade A cannot readily be identified in our current state of knowledge. Further studies using high-throughput sequencing and including other still unsampled species in this clade will help assess how far genetic data alone can help reveal the true levels of species diversity in this part of the phylogeny, but it is likely that adding more data will make the identification of diagnostic, monophyletic groups more – rather than less – difficult.

Lack of diagnosability affects most nodes in the Ipomoeeae phylogeny, not only clade A. In order to find *diagnosable* clades, we need to look at much smaller groups of species, such as the Arborescens clade (a group of 10 species with a tree habit in clade A1) or the Quamoclit clade (16 species with subapically awned sepals, some of which were treated in the past as a different genus *Quamoclit* L., now in clade B2) (Wood & al., 2020). In the African grade (Figs. 1, 3), the Astripomoea clade, which includes 15 species of former *Astripomoea* and *Ipomoea*, can be recognised by the presence of stellate hairs and a somewhat elongate stigma, although both characters appear independently in several American species from other clades. Similarly, the clade including members of the former genus *Stictocardia*, which intermingle with several *Ipomoea* species, can be diagnosed by the accrescent sepals and the glandular abaxial leaf surface, yet neither character is unique to this clade. These small clades form part of bigger clades that cannot be readily diagnosed, and neighbouring clades are also not diagnosable.

Thus, the hypothetical recognition of smaller clades as independent genera hinted at by recent authors (Eserman & al., 2020; Staples & al., 2021) would necessarily lead to the generation of non-diagnosable taxa, and would certainly affect completeness, particularly if an attempt was made to achieve diagnosability. Furthermore, the inclusion of more species in the molecular phylogenies (c. 30% of Ipomoeeae species have not been sequenced yet) will likely further complicate the reconciliation of these properties. Additionally, the morphological *continuum* and high levels of homoplasy that characterise the tribe Ipomoeeae also advise against trying to split *Ipomoea* into smaller non-diagnosable genera. Ignoring this, and thus persevering in the use of artificial groups – as opposed to natural or monophyletic groups –, will result in repeating the problems of previous classifications of *Ipomoea* that were to a degree unnatural, with many groups neither monophyletic nor well-defined (McDonald, 1995; Miller & al., 2004). In the longer term, the recognition of artificial taxa or the publication of comparative studies with misleading results based on a very limited taxon sampling (Baker & al., 2022; Rattanakrajang & al., 2022; Simões & al., 2022) will hindera good understanding of the diversity and evolution of the group.

### Option 3. Recognise an expanded *Ipomoea*

With the evidence available, we consider the third option, recognising an expanded *Ipomoea*, to be the most appropriate solution. All phylogenetic analyses to date have shown Ipomoeeae is a monophyletic group with high support (McDonald & Mabry, 1992; Miller & al., 1999; Wilkin, 1999; Stefanović & al., 2002, 2003; Eserman & al., 2014; Muñoz-Rodríguez & al., 2019). This clade includes all members of the family Convolvulaceae with echinate, pantoporate (spiny) pollen – a synapomorphy in the family. Thus, an expanded *Ipomoea* that incorporates all segregate genera meets the four properties outlined above: *monophyly, resolution, diagnosability* and *completeness*.

Incorporating all segregate genera into *Ipomoea* requires fewer nomenclatural changes than splitting *Ipomoea* into multiple smaller genera. The number of species in the segregate genera currently recognised is c. 174 – many of which originally described in *Ipomoea* – and, as explained above, most of them belong to the Old World clade, a clade that nevertheless contains more species of *Ipomoea* in the traditional sense than of all other genera combined. The alternative of splitting *Ipomoea* into several smaller genera would require changing approximately 500 *Ipomoea* names, a solution certainly less favourable to nomenclatural stability. In addition, recognising an expanded *Ipomoea* would also avoid nomenclatural changes affecting species of economic interest, not only the two crop species, sweet potato (*I. batatas*) and water spinach (*I. aquatica*), but also the many ornamental species that belong to different clades. Finally, recognising an expanded *Ipomoea* as the sole genus in Ipomoeeae would also make the proposal to change the type of the genus (Eserman & al., 2020) unnecessary.

Some authors could argue that the different clades in an expanded genus *Ipomoea* could be formally named following a Linnaean classification system, for example at subgeneric rank. Although we have no theoretical objection to the naming of these cryptic nodes as subgenera, sections, etc., we think there is little benefit in establishing a complex named hierarchy of non-diagnosable taxa that would be of little practical use. No less important, we believe that any attempt to provide an infrageneric classification of *Ipomoea* following a traditional Linnaean model is bound to be artificial, impractical and doomed to failure (Carine & Scotland, 2002). It is difficult, or impossible, to achieve an infrageneric classification (or a generic classification if *Ipomoea* is split) in which the properties of monophyly, resolution, diagnosability, and completeness are met, especially considering the poor current knowledge of many species.

In the delimitation we propose, *Ipomoea* splits into two main clades (Fig. 3): one clade (Old World clade) including slightly less than half of the species in the genus, mostly restricted to Africa and Asia, and another clade formed by a grade of African species and a species-rich clade dominated by American species (New World clade). Several clades are recognised and strongly supported in all phylogenies. Some of these clades have diagnostic morphological characters, for example species with coriaceous sepals (clade A2) or species with a tree habit (a small clade within A1), but most clades are cryptic. With this in mind, in 2019 we did not attempt any formal recognition of infrageneric ranks in *Ipomoea* and still prefer to refer to the genus as a whole while using informal names to refer to specific parts of the phylogeny, in line with the approach followed for example in *Solanum*. Nevertheless, we acknowledge that using letters to refer to the main clades in the phylogeny may not be satisfactory, and thus in Figs. 1 and 3 we suggest informal names alongside the letters we used in our previous works.

## Conclusion

Although focused on *Ipomoea*, the discussion in this paper highlights the more general tension that exists in contemporary systematics between phylogenetics and taxonomy. At one extreme, this tension has resulted in proposals for a “phylocode” that is rank-free classification based entirely on phylogeny (De Queiroz & Gauthier, 1992; Cantino & al., 2020). The reasons why the PhyloCode has not been widely adopted are numerous but include the fact that most authors consider classifications should reflect phylogeny, but that *classification* and *phylogeny* are distinct. Classifications are viewed as useful information retrieval systems for diagnosable monophyletic groups and, although they reflect aspects of phylogeny, their role is distinct. At the other extreme there are colleagues, albeit few now, that prefer to explicitly argue against monophyly in favour of paraphyly (Brummitt, 2008; Gilbert, 2020) or implicitly support classifications that include non-monophyletic taxa (e.g., *Begonia*, former Ipomoeeae). In between these two extremes is the mainstream of systematics but here too the same tensions in the relationship between classification and phylogeny manifest themselves in numerous ways. These issues will surely increase as molecular sequence data achieve more phylogenetic resolution. It is in this context that we as a community need to be clear about what we consider is the general purpose of a classification.

Our proposal to recognise an expanded *Ipomoea* reconciles the properties of monophyly, resolution, diagnosability, and completeness, as well as favours nomenclatural stability, and is in line with what has been agreed in other megadiverse plant genera. As we have shown, in all these cases splitting big genera into smaller units, be it genera, subgenera, or sections, appears to be problematic if the aim is to reconcile monophyly and diagnosability, and thus authors have often preferred to recognise an expanded, monophyletic and diagnosable genus - regardless of its size. In the circumscription we propose, *Ipomoea* is the only genus in the tribe Ipomoeeae and includes almost half of the species in the family. Most nomenclatural changes necessary to transfer species from the segregate genera to *Ipomoea* have already been published (Muñoz-Rodríguez & al., 2019; Wood & al., 2022).

In our studies of *Ipomoea*, we have continually prioritised species-level taxonomic accounts as we consider these the priority output for taxonomy, given how little we know about individual species (Scotland & Wood, 2012; Goodwin & al., 2020). A secondary but useful output is the fact that we now have comprehensively sampled phylogenies that can be used for a range of evolutionary studies (Muñoz-Rodríguez & al., 2018, 2019, 2022a; Carruthers, 2019; Carruthers & al., 2020a,b). Taking these phylogenies into account forms a vital part in sorting (classifying) the species in a diagnosable, monophyletic genus within their evolutionary context.

## Supplementary Material

Supplementary Figures

## References

[R1] Abdel Khalik K (2013). Systematic implications of seed coat diversity in some representatives of the genus *Ipomoea* (Convolvulaceae). Turk J Bot.

[R2] Abdel Khalik K, Osman G, Al-Amoudi W (2012). Genetic diversity and taxonomic relationships of some *Ipomoea* species based on analysis of RAPD-PCR and SDS-PAGE of seed proteins. Austral J Crop Sci.

[R3] Austin DF (1975). Flora of Panama: Family 164. Convolvulaceae. Ann Missouri Bot Gard.

[R4] Austin DF (1982). Flora of Ecuador, vol. 15(165), Convolvulaceae.

[R5] Austin DF (1991). *Ipomoea littoralis* (Convolvulaceae) – Taxonomy, distribution, and ethnobotany. Econ Bot.

[R6] Austin DF (1997). Dissolution of *Ipomoea* series *Anisomerae* (Convolvulaceae). J Torrey Bot Soc.

[R7] Austin DF (2013). Moon-flower (*Ipomoea alba*, Convolvulaceae)— Medicine, rubber enabler, and ornamental: A review. Econ Bot.

[R8] Austin DF, McDonald JA (2014). Relationships and taxonomy of *Ipomoea variabilis* (Convolvulaceae). Phytoneuron.

[R9] Austin DF, Staples GW (1991). A revision of the Neotropical species of *Turbina* Raf. (Convolvulaceae). Bull Torr Bot Club.

[R10] Backlund A, Bremer K (1998). To be or not to be – Principles of classification and monotypic plant families. Taxon.

[R11] Baker WJ, Bailey P, Barber V, Barker A, Bellot S, Bishop D, Botigué LR, Brewer G, Carruthers T, Clarkson JJ, Cook J (2022). A comprehensive phylogenomic platform for exploring the Angiosperm Tree of Life. Syst Biol.

[R12] Bohs L, Keating RC, Hollowell VC, Croat TB (2005). A Festschrift for William G D’arcy: The legacy of a taxonomist Monographs in Systematic Botany from the Missouri Botanical Garden.

[R13] Bohs L, Olmstead RG (1997). Phylogenetic relationships in *Solanum* (Solanaceae) based on *ndhF* sequences. Syst Bot.

[R14] Bouman RW, Keßler PJA, Telford IRH, Bruhl JJ, Strijk JS, Saunders RMK, Welzen PC (2021). Molecular phylogenetics of *Phyllanthus* sensu lato (Phyllanthaceae): Towards coherent monophyletic taxa. Taxon.

[R15] Bremekamp CEB (1944). Materials for a monograph of the Strobilanthinae (Acanthaceae). Verh Kon Ned Akad Wetensch, Afd Natuurk, Sect 2.

[R16] Brummitt RK (1992). Vascular plant families and genera: A listing of the genera of vascular plants of the world according to their families, as recognised in the Kew Herbarium; with An analysis of relationships of the flowering plant families according to eight systems of classification.

[R17] Brummitt RK, Van der Maesen LJG, Van der Burgt XM, Van Medenbach de Rooy JM (1996). The biodiversity of African plants.

[R18] Brummitt RK (2008). Evolution in taxonomic perspective. Taxon.

[R19] Bruyns PV, Mapaya RJ, Hedderson TJ (2006). A new subgeneric classification for *Euphorbia* (Euphorbiaceae) in southern Africa based on ITS and *psbA-trnH* sequence data. Taxon.

[R20] Bruyns PV, Klak C, Hanáček P (2017). A revised, phylogenetically-based concept of *Ceropegia* (Apocynaceae). S African J Bot.

[R21] Bryant HN (1994). Comments on the phylogenetic definition of taxon names and conventions regarding the naming of crown clades. Syst Biol.

[R22] Cantino PD, Olmstead RG, Wagstaff SJ (1997). A comparison of phylogenetic nomenclature with the current system: A botanical case study. Syst Biol.

[R23] Cantino PD, De Queiroz K (2020). International code of phylogenetic nomenclature: PhyloCode, Version 6.

[R24] Carine MA, Scotland RW (2002). Classification of Strobilanthinae (Acanthaceae): Trying to classify the unclassifiable?. Taxon.

[R25] Carruthers J, Robin L (2010). Taxonomic imperialism in the battles for *Acacia*: Identity and science in South Africa and Australia. Trans Roy Soc South Africa.

[R26] Carruthers T (2019). What can we learn about plant evolution from a robust phylogenetic framework?.

[R27] Carruthers T, Muñoz-Rodríguez P, Wood JRI, Scotland RW (2020a). The temporal dynamics of evolutionary diversification in *Ipomoea*. Molec Phylogen Evol.

[R28] Carruthers T, Sanderson MJ, Scotland RW (2020b). The implications of lineage-specific rates for divergence time estimation. Syst Biol.

[R29] Choisy JD (1834). Convolvulaceae Orientales. Mém Soc Phys Genève.

[R30] Choisy JD (1838). De Convolvulaceis. Mém Soc Phys Genève.

[R31] Choisy JD, Candolle A (1845). Prodromus systematis naturalis regni vegetabilis.

[R32] Claßen-Bockhoff R, Speck T, Tweraser E, Wester P, Thimm S, Reith M (2004). The staminal lever mechanism in *Salvia* L. (Lamiaceae): A key innovation for adaptive radiation?. Organisms Diversity Evol.

[R33] Das S (2011). Congruence between morphological and molecular approach in understanding species relationship in *Ipomoea* spp.: A rare event in taxonomy. Asian J Pl Sci.

[R34] De Queiroz K, Gauthier J (1992). Phylogenetic taxonomy. Annual Rev Ecol Syst.

[R35] Domínguez Lozano F, Schwartz MW (2005). Patterns of rarity and taxonomic group size in plants. Biol Conservation.

[R36] Doorenbos J, Sosef MSM, de Wilde JJFE (1998). The sections of Begonia including descriptions, keys and species lists. Studies in Begoniaceae 4.

[R37] Dorsey BL, Haevermans T, Aubriot X, Morawetz JJ, Riina R, Steinmann VW, Berry PE (2013). Phylogenetics, morphological evolution, and classification of *Euphorbia* subgenus *Euphorbia*. Taxon.

[R38] Drew BT, González-Gallegos JG, Xiang C-L, Kriebel R, Drummond CP, Walker JB, Sytsma KJ (2017). *Salvia* united: The greatest good for the greatest number. Taxon.

[R39] Eserman LA, Tiley GP, Jarret RL, Leebens-Mack JH, Miller RE (2014). Phylogenetics and diversification of morning glories (tribe Ipomoeeae, Convolvulaceae) based on whole plastome sequences. Amer J Bot.

[R40] Eserman LA, Sosef MSM, Simão-Bianchini R, Utteridge TMA, Barbosa JCJ, Buril MT, Chatrou LW, Clay K, Delgado G, Desquilbet TE, Ferreira PPA (2020). (2786) Proposal to change the conserved type of *Ipomoea*, nom. cons. (Convolvulaceae). Taxon.

[R41] Fazekas AJ, Kesanakurti PR, Burgess KS, Percy DM, Graham SW, Barrett SCH, Newmaster SG, Hajibabaei M, Husband BC (2009). Are plant species inherently harder to discriminate than animal species using DNA barcoding markers?. Molec Ecol Resources.

[R42] Folorunso AE (2013). Taxonomic evaluation of fifteen species of *Ipomoea* L. (Convolvulaceae) from South-Western Nigeria using foliar micromorphological characters. Notul Sci Biol.

[R43] Forrest LL, Hollingsworth PM (2003). A recircumscription of *Begonia* based on nuclear ribosomal sequences. Pl Syst Evol.

[R44] Fragoso-Martínez I, Martínez-Gordillo M, Salazar GA, Sazatornil F, Jenks AA, del García Peña MR, Barrera-Aveleida G, Benitez-Vieyra S, Magallón S, Cornejo-Tenorio G, Granados Mendoza C (2018). Phylogeny of the Neotropical sages (*Salvia* subg. *Calosphace*; Lamiaceae) and insights into pollinator and area shifts. Pl Syst Evol.

[R45] Frodin DG (2004). History and concepts of big plant genera. Taxon.

[R46] Frohlich MW, Sage RF, Craven LA, Schuster S, Gigot G, Hilger HH, Akhani H, Mahdavi P, Luebert F, Weigend M, Thulin M (2022). Molecular phylogenetics of *Euploca* (Boraginaceae): Homoplasy in many characters, including the C_4_ photosynthetic pathway. Bot J Linn Soc.

[R47] Gagnon E, Hilgenhof R, Orejuela A, McDonnell A, Sablok G, Aubriot X, Giacomin L, Gouvêa Y, Bragionis T, Stehmann JR, Bohs L (2022). Phylogenomic discordance suggests polytomies along the backbone of the large genus Solanum. Amer J Bot.

[R48] Gilbert MG, Goyder DJ, Golbert MG, Ventner HJT (2020). Flora Zambesiaca.

[R49] Global Carex Group (2015). Making *Carex* monophyletic (Cyperaceae, tribe Cariceae): A new broader circumscription. Bot J Linn Soc.

[R50] González-Gallegos JG, Bedolla-García BY, Cornejo-Tenorio G, Fernández-Alonso JL, Fragoso-Martínez I, del García-Peña MR, Harley RM, Klitgaard B, Martínez-Gordillo MJ, Wood JRI, Zamudio S (2020). Richness and distribution of *Salvia* subg. *Calosphace* (Lamiaceae). Int J Pl Sci.

[R51] Goodwin ZA, Muñoz-Rodríguez P, Harris DJ, Wells T, Wood JRI, Filer D, Scotland RW (2020). How long does it take to discover a species?. Syst Biodivers.

[R52] Gray A (1878). Synoptical flora of North America.

[R53] Grisebach AHR (1864). Flora of the British West Indian islands.

[R54] Hallier H (1894). Convolvulaceae Africanae. Bot Jahrb Syst.

[R55] Hennig W (1966). Phylogenetic systematics.

[R56] Hoffmann P, Kathriarachchi H, Wurdack KJ (2006). A phylogenetic classification of Phyllanthaceae (Malpighiales: Euphorbiaceae sensu lato). Kew Bull.

[R57] Hörandl E, Stuessy TF (2010). Paraphyletic groups as natural units of biological classification. Taxon.

[R58] Horn JW, Van Ee BW, Morawetz JJ, Riina R, Steinmann VW, Berry PE, Wurdack KJ (2012). Phylogenetics and the evolution of major structural characters in the giant genus *Euphorbia* L. (Euphorbiaceae). Molec Phylogen Evol.

[R59] House HD (1908). The North American species of the genus *Ipomoea*. Ann New York Acad Sci.

[R60] Hunziker AT (2001). Genera Solanacearum: The genera of Solanaceae illustrated, arranged according to a new system.

[R61] IPNI (2021). International Plant Names Index.

[R62] Jara E, Muñoz-Rodríguez P, Wood JRI, Beltrán H (2020). *Ipomoea noemana* (Convolvulaceae) a new species from Ancash eastern slope in Peru. Phytotaxa.

[R63] Jenks AA, Walker JB, Kim S-C (2013). Phylogeny of New World *Salvia* subgenus *Calosphace* (Lamiaceae) based on cpDNA (*psbA-trnH*) and nrDNA (ITS) sequence data. J Pl Res.

[R64] Johnson RW (1986). Four new species of *Ipomoea* L. (Convolvulaceae) from Australia. Austrobaileya.

[R65] Kathriarachchi H, Hoffmann P, Samuel R, Wurdack KJ, Chase MW (2005). Molecular phylogenetics of Phyllanthaceae inferred from five genes (plastid *atpB, matK*, 3′*ndhF, rbcL*, and nuclear *PHYC*. Molec Phylogen Evol.

[R66] Lawand PR, Shimpale VB (2021). *Argyreia sharadchandrajii* (Convolvulaceae), a new species from the Western Ghats, India. Rheedea.

[R67] Linnaeus C (1753). Species plantarum.

[R68] Mabberley DJ (2017). Mabberley’s plant-book: A portable dictionary of plants, their classification and uses.

[R69] Manos PS, Miller RE, Wilkin P (2001). Phylogenetic analysis of *Ipomoea, Argyreia, Stictocardia*, and *Turbina* suggests a generalized model of morphological evolution in morning glories. Syst Bot.

[R70] Marderosian AHD (1965). Nomenclatural history of the morning glory, *Ipomoea violacea* (L). Taxon.

[R71] McDonald JA (1995). Revision of *Ipomoea* section *Leptocallis* (Convolvulaceae). Harvard Pap Bot.

[R72] McDonald JA, Austin DF (1990). Changes and additions in *Ipomoea* sect. Batatas Brittonia.

[R73] McDonald JA, Mabry TJ (1992). Phylogenetic systematics of New World *Ipomoea* (Convolvulaceae) based on chloroplast DNA restriction site variation. Pl Syst Evol.

[R74] Meeuse ADJ, Welman WG (2000). Flora of Southern Africa, vol. 28(1), Convolvulaceae.

[R75] Miller RE, Rausher MD, Manos PS (1999). Phylogenetic systematics of *Ipomoea* (Convolvulaceae) based on ITS and *Waxy* sequences. Syst Bot.

[R76] Miller RE, McDonald JA, Manos PS (2004). Systematics of *Ipomoea* subgenus *Quamoclit* (Convolvulaceae) based on ITS sequence data and a Bayesian phylogenetic analysis. Amer J Bot.

[R77] Moonlight PW, Ardi WH, Padilla LA, Chung K-F, Fuller D, Girmansyah D, Hollands R, Jara-Muñoz A, Kiew R, Leong W-C, Liu Y (2018). Dividing and conquering the fastest-growing genus: Towards a natural sectional classification of the mega-diverse genus *Begonia* (Begoniaceae). Taxon.

[R78] Muñoz-Rodríguez P (2019). Systematic studies of the sweet potato and its wild relatives.

[R79] Muñoz-Rodríguez P, Carruthers T, Wood JRI, Williams BRM, Weitemier K, Kronmiller B, Ellis D, Anglin NL, Longway L, Harris SA, Rausher MD (2018). Reconciling conflicting phylogenies in the origin of sweet potato and dispersal to Polynesia. Curr Biol.

[R80] Muñoz-Rodríguez P, Carruthers T, Wood JRI, Williams BRM, Weitemier K, Kronmiller B, Goodwin Z, Sumadijaya A, Anglin NL, Filer D, Harris D (2019). A taxonomic monograph of Ipomoea integrated across phylogenetic scales. Nature Pl.

[R81] Muñoz-Rodríguez P, Wells T, Wood JRI, Carruthers T, Anglin NL, Jarret RL, Scotland RW (2022a). Discovery and characterisation of sweetpotato’s closest tetraploid relative. New Phytol.

[R82] Muñoz-Rodríguez P, Wood JRI, Scotland RW, Monro AK, Mayo SJ (2022b). Cryptic species: Morphological stasis, circumscription and hidden diversity.

[R83] O’Donell C (1960). Notas sobre convolvuláceas americanas. Lilloa.

[R84] Ogunwenmo KO (2008). Evolutionary and taxonomic studies of *Ipomoea* L. sect. *Involucratae* Bak., Randle (Convolvulaceae) in Nigeria. Feddes Repert.

[R85] Olmstead RG, Palmer JD (1997). Implications for the phylogeny, classification, and biogeography of *Solanum* from cpDNA restriction site variation. Syst Bot.

[R86] Peirson JA, Bruyns PV, Riina R, Morawetz JJ, Berry PE (2013). A molecular phylogeny and classification of the largely succulent and mainly African *Euphorbia* subg. *Athymalus* (Euphorbiaceae). Taxon.

[R87] POWO (2020). Plants of the World Online [Database].

[R88] Pruesapan K, Telford IRH, Bruhl JJ, Draisma SGA, Van Welzen PC (2008). Delimitation of *Sauropus* (Phyllanthaceae) based on plastid *matK* and nuclear ribosomal ITS DNA sequence data. Ann Bot (Oxford).

[R89] Rasingam L, Swamy J (2020). *Brachystelma telanganense* (Apocynaceae: Asclepiadoideae-Ceropegieae) – A new species from Telangana, India. Rheedea.

[R90] Rattanakrajang P, Sumanon P, Traiperm Staples G, Utteridge T (2022). Reduction of *Blinkworthia* (Convolvulaceae) based on multilocus phylogenetic reconstruction and resurrection of a species from synonymy revealed by phenetic analyses. Kew Bull.

[R91] RBG Kew (2016). The state of the world’s plants report – 2016.

[R92] Riina R, Peirson JA, Geltman DV, Molero J, Frajman B, Pahlevani A, Barres L, Morawetz JJ, Salmaki Y, Zarre S, Kryukov A (2013). A worldwide molecular phylogeny and classification of the leafy spurges, *Euphorbia* subgenus *Esula* (Euphorbiaceae). Taxon.

[R93] Sampathkumar R (1979). Karyomorphological studies in some South Indian Convolvulaceae. Cytologia.

[R94] Sanderson MJ, Wojciechowski MF (1996). Diversification rates in a temperate legume clade: Are there “so many species” of Astragalus (Fabaceae)?. Amer J Bot.

[R95] Santos D, Alencar J, Bezerra Loiola MI, Buril MT (2020). *Ipomoea bonsai* (Convolvulaceae), a magnificent new species from the Caatinga domain, Brazil. Syst Bot.

[R96] Santos D, Souza EB, Buril MT (2021). *Ipomoea lanifolia* sp. nov. (Convolvulaceae), a new species endemic to the Ibiapaba plateau in northeastern Brazil. Rodriguésia.

[R97] Särkinen T, Bohs L, Olmstead RG, Knapp S (2013). A phylogenetic framework for evolutionary study of the nightshades (Solanaceae): A dated 1000-tip tree. B M C Evol Biol.

[R98] Sayers E, Cavanaugh M, Clark K, Pruitt KD, Schoch CL, Sherry ST, Karsch-Mizrachi I (2021). GenBank. Nucl Acids Res.

[R99] Scotland RW, Sanderson MJ (2004). The significance of few versus many in the Tree of Life. Science.

[R100] Scotland RW, Wood JRI (2012). Accelerating the pace of taxonomy. Trends Ecol Evol.

[R101] Simões ARG, Eserman LA, Zuntini AR, Chatrou LW, Utteridge TMA, Maurin O, Rokni S, Roy S, Forest F, Baker WJ, Stefanović S (2022). A bird’s eye view of the systematics of Convolvulaceae: Novel insights from nuclear genomic data. Frontiers Pl Sci (Online journal).

[R102] Spooner DM, Anderson GJ, Jansen RK (1993). Chloroplast DNA evidence for the interrelationships of tomatoes, potatoes, and pepinos (Solanaceae. Amer J Bot.

[R103] Sprengel CK (1793). Das entdeckte Geheimnis der Natur im Bau und in der Befruchtung der Blumen.

[R104] Staples GW, Traiperm P (2017). A nomenclatural review of *Argyreia* (Convolvulaceae). Taxon.

[R105] Staples GW, Wiersema JH, Chambers NA, Austin DF (2005). The restoration of *Ipomoea muricata* (L.) Jacq. (Convolvulaceae). Taxon.

[R106] Staples GW, Traiperm P, Sugau JB, Pornpongrungrueng P (2014). *Ipomoea cambodiensis* Gagnep., Courchet (Convolvulaceae) recharacterised with notes on its distribution and ecology. Adansonia.

[R107] Staples GW, Chitchak N, Kochaiphat P, Rattamanee C, Rattanakrajang P, Traiperm P (2021). Convolvulaceae in the Flora of Thailand: Addenda, corrigenda and emendanda, I. Thai Forest Bull, Bot.

[R108] Stefanović S, Krueger L, Olmstead RG (2002). Monophyly of the Convolvulaceae and circumscription of their major lineages based on DNA sequences of multiple chloroplast loci. Amer J Bot.

[R109] Stefanović S, Austin DF, Olmstead RG (2003). Classification of Convolvulaceae: A phylogenetic approach. Syst Bot.

[R110] Steinmann VW, Porter JM (2002). Phylogenetic relationships in Euphorbieae (Euphorbiaceae) based on ITS and *ndhF* sequence data. Ann Missouri Bot Gard.

[R111] Stevens PF (2001). Angiosperm Phylogeny Website, version 14.

[R112] Swamy J, Ramana PV (2018). Notes on the taxonomy and distribution of the Bengal morning glory *Ipomoea rubens* Choisy (Convolvulaceae) in India. J Threat Taxa.

[R113] Swenson NG (2009). Phylogenetic resolution and quantifying the phylogenetic diversity and dispersion of communities. PLoS ONE.

[R114] Thulin M (2003). Notes on *Convolvulus, Astripomoea, Ipomoea* and *Merremia* (Convolvulaceae) from the Horn of Africa. Nordic J Bot.

[R115] Ting YC, Kehr AE (1953). Meiotic studies in the sweet potato (*Ipomoea batatas* Lam). J Heredity.

[R116] Ting YC, Kehr AE, Miller JC (1957). A cytological study of the sweet potato plant *Ipomoea batatas* (L.) Lam. and its related species. Amer Naturalist.

[R117] Traiperm P, Fujikawa K, Chitchak N, Srisanga P, Maknoi C, Staples G (2019). A new species of *Argyreia* (Convolvulaceae) from Myanmar. Willdenowia.

[R118] Traiperm P, Suddee S (2020). A new species of *Argyreia* (Convolvulaceae) from Thailand. PhytoKeys.

[R119] Turland N, Wiersema J, Barrie F, Greuter W, Hawksworth D, Herendeen P, Knapp S, Kusber W-H, Li D-Z, Marhold K, May T (2018). International Code of Nomenclature for algae, fungi, and plants Regnum Vegetabile 159.

[R120] Van Ooststroom SJ (1940). The Convolvulaceae of Malaysia, III. Blumea.

[R121] Van Ooststroom SJ, Van Steenis CGGJ (1953). Flora Malesiana, ser I.

[R122] Van Welzen PC, Pruesapan K, Telford IRH, Esser H-J, Bruhl JJ (2014). Phylogenetic reconstruction prompts taxonomic changes in *Sauropus, Synostemon* and *Breynia* (Phyllanthaceae tribe Phyllantheae). Blumea.

[R123] Verdcourt B (1963). Flora of Tropical East Africa: Convolvulaceae.

[R124] Vij SP, Singh S, Sachdeva VP (1977). Cytomorphological studies in Convolvulaceae. II. *Ipomoea* and allied genera. Cytologia.

[R125] Walker JB, Sytsma KJ (2007). Staminal evolution in the genus *Salvia* (Lamiaceae): Molecular phylogenetic evidence for multiple origins of the staminal lever. Ann Bot (Oxford).

[R126] Walker JB, Sytsma KJ, Treutlein J, Wink M (2004). *Salvia* (Lamiaceae) is not monophyletic: Implications for the systematics, radiation, and ecological specializations of *Salvia* and tribe Mentheae. Amer J Bot.

[R127] Weese TL, Bohs L (2007). A three-gene phylogeny of the genus *Solanum* (Solanaceae). Syst Bot.

[R128] Wilkin P (1999). A morphological cladistic analysis of the *Ipomoeeae* (Convolvulaceae). Kew Bull.

[R129] Will M, Schmalz N, Classen-Bockhoff R (2015). Towards a new classification of *Salvia* s.l.: (re)establishing the genus Pleudia Raf. Turk J Bot.

[R130] Willis JC (1922). Age and area: A study in geographical distribution and origin of species.

[R131] Willis JC, Yule GU (1922). Some statistics of evolution and geographical distribution in plants and animals, and their significance. Nature.

[R132] Wood JRI, Scotland RW (2017a). Misapplied names, synonyms and new species of *Ipomoea* (Convolvulaceae) from South America. Kew Bull.

[R133] Wood JRI, Scotland RW (2017b). Notes on *Ipomoea* (Convolvulaceae) from the Amazonian periphery. Kew Bull.

[R134] Wood JRI, Scotland RW (2017c). Notes on *Ipomoea* L. (Convolvulaceae) in Cuba and neighbouring islands with a checklist of species found in Cuba. Kew Bull.

[R135] Wood JRI, Carine MA, Harris D, Wilkin P, Williams BRM, Scotland RW (2015). *Ipomoea* (Convolvulaceae) in Bolivia. Kew Bull.

[R136] Wood JRI, de Arrúa RD, de Rojas GD, Scotland RW (2016a). Two overlooked species of *Ipomoea* L. (Convolvulaceae) from Paraguay. Kew Bull.

[R137] Wood JRI, Urbanetz C, Scotland RW (2016b). *Ipomoea pantanalensis*, a new species of *Ipomoea* L. (Convolvulaceae) from the Pantanal, Brazil. Kew Bull.

[R138] Wood JRI, Buril MT, Scotland RW (2017a). Remarkable disjunctions in *Ipomoea* species (Convolvulaceae) from NE Brazil and Central America and their taxonomic implications. Kew Bull.

[R139] Wood JRI, Muñoz-Rodríguez P, Degen R, Scotland RW (2017b). New species of *Ipomoea* (Convolvulaceae) from South America. PhytoKeys.

[R140] Wood JRI, Vasconcelos LV, Simão-Bianchini R, Scotland RW (2017c). New species of *Ipomoea* (Convolvulaceae) from Bahia. Kew Bull.

[R141] Wood JRI, Martinez Ugarteche MT, Muñoz-Rodríguez P, Scotland RW (2018). Additional notes on *Ipomoea* (Convolvulaceae) in Bolivia. Kew Bull.

[R142] Wood JRI, Muñoz-Rodríguez P, Williams BRM, Scotland RW (2020). A foundation monograph of *Ipomoea* (Convolvulaceae) in the New World. PhytoKeys.

[R143] Wood JRI, Muñoz-Rodríguez P, Scotland RW (2022). Priorities for taxonomic studies of Indian Convolvulaceae. J Econ Taxon Bot.

[R144] Wright J (2015). The naming of the shrew: A curious history of Latin names.

[R145] Wurdack KJ, Hoffmann P, Chase MW (2005). Molecular phylogenetic analysis of uniovulate Euphorbiaceae (Euphorbiaceae sensu stricto) using plastid *RBCL* and *TRNL-F* DNA sequences. Amer J Bot.

[R146] Yang Y, Riina R, Morawetz JJ, Haevermans T, Aubriot X, Berry PE (2012). Molecular phylogenetics and classification of *Euphorbia* subgenus *Chamaesyce* (Euphorbiaceae). Taxon.

