# Supplementary figures and images for: The challenges of classifying big genera such as Ipomoea
Source: Taxon. Author manuscript; Available in PMC 2025 Jul 20. (PMC7617906; doi:10.1002/tax.12887)

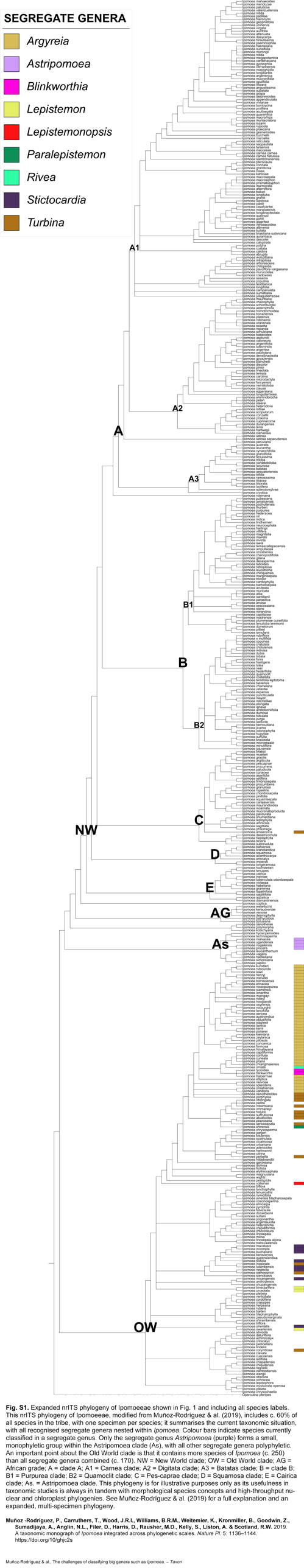

Supplement: Supplementary Figures [file EMS206638-supplement-Supplementary_Figures.zip › tax12887-sup-0001-figures1.jpg]

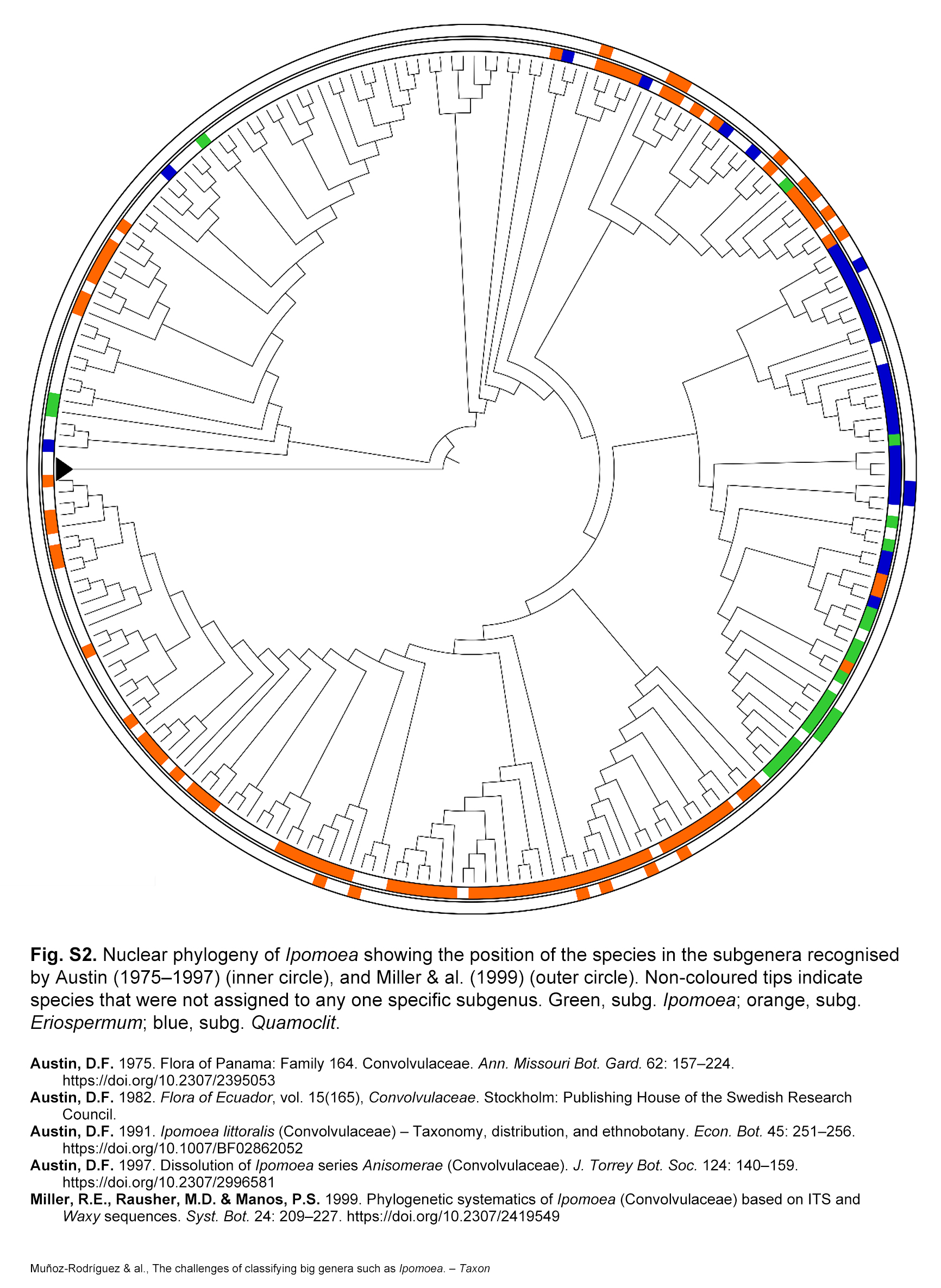

Supplement: Supplementary Figures [file EMS206638-supplement-Supplementary_Figures.zip › tax12887-sup-0002-figures2.jpg]
